# Diagnostic System for Rapid and Sensitive Differential Detection of Pathogens

**DOI:** 10.3201/eid1102.040492

**Published:** 2005-02

**Authors:** Thomas Briese, Gustavo Palacios, Mark Kokoris, Omar Jabado, Zhiqiang Liu, Neil Renwick, Vishal Kapoor, Inmaculada Casas, Francisco Pozo, Ron Limberger, Pilar Perez-Brena, Jingyue Ju, W. Ian Lipkin

**Affiliations:** * Columbia University, New York, New York, USA;; † Qiagen Inc., Valencia, California, USA;; ‡ Instituto de Salud Carlos III, Majadahonda, Madrid, Spain;; § New York State Department of Health, Albany, New York, USA

**Keywords:** Mass spectroscopy, PCR, multiplex, differential diagnosis, infectious diseases

## Abstract

Naturally emerging and deliberately released pathogens demand new detection strategies to allow early recognition and containment. We describe a diagnostic system for rapid, sensitive, multiplex discrimination of microbial gene sequences and report its application for detecting 22 respiratory pathogens in clinical samples.

Efficient laboratory diagnosis of infectious diseases is increasingly important to clinical management and public health. Methods to directly detect nucleic acids of microbial pathogens in clinical specimens are rapid, sensitive, and may succeed when culturing the organism fails. Clinical syndromes are infrequently specific for single pathogens; thus, assays are needed that allow multiple agents to be simultaneously considered. Current multiplex assays employ gel-based formats in which products are distinguished by size, fluorescent reporter dyes that vary in color, or secondary enzyme hybridization assays. Gel-based assays are reported that detect 2–8 different targets with sensitivities of 2–100 PFU or <1–5 PFU, depending on whether amplification is carried out in a single or nested format, respectively (*1**–**4*). Fluorescence reporter systems achieve quantitative detection with sensitivity similar to that of nested amplification; however, their capacity to simultaneously query multiple targets is limited to the number of fluorescent emission peaks that can be unequivocally resolved. At present, up to 4 fluorescent reporter dyes can be detected simultaneously (*5**,**6*). Multiplex detection of up to 9 pathogens has been achieved in hybridization enzyme systems; however, the method requires cumbersome postamplification processing (*7*).

## The Study

**Figure 1:**
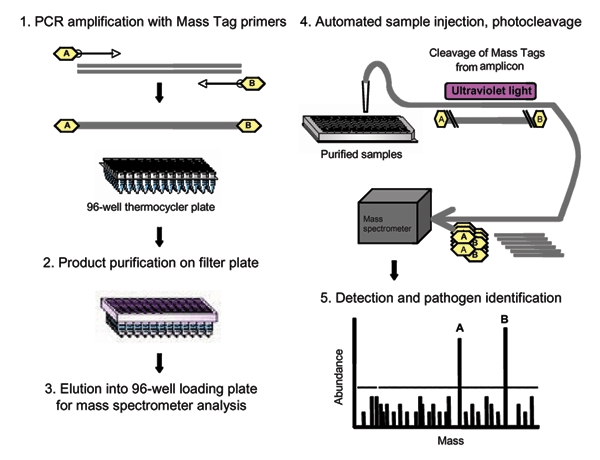
Schematic representation of Mass Tag polymerase chain reaction (PCR).

To address the need for sensitive multiplex assays in diagnostic molecular microbiology, we created a polymerase chain reaction (PCR) platform in which microbial gene targets are coded by a library of 64 distinct Masscode tags (Qiagen Masscode technology, Qiagen, Hilden, Germany). A schematic representation of this approach is shown in Figure 1. Microbial nucleic acids (RNA, DNA, or both) are amplified by multiplex reverse transcription (RT)-PCR using primers labeled by a photocleavable link to molecular tags of different molecular weight. After removing unincorporated primers, tags are released by UV irradiation and analyzed by mass spectrometry. The identity of the microbe in the clinical sample is determined by its cognate tags.

As a first test of this technology, we focused on respiratory disease because differential diagnosis is a common clinical challenge, with implications for outbreak control and individual case management. Multiplex primer sets were designed to identify up to 22 respiratory pathogens in a single Mass Tag PCR reaction; sensitivity was established by using synthetic DNA and RNA standards as well as titered viral stocks; the utility of Mass Tag PCR was determined in blinded analysis of previously diagnosed clinical specimens.

Oligonucleotide primers were designed in conserved genomic regions to detect the broadest number of members for a given pathogen species by efficiently amplifying a 50- to 300-bp product. In some instances, we selected established primer sets; in others, we used a software program designed to cull sequence information from GenBank, perform multiple alignments, and maximize multiplex performance by selecting primers with uniform melting temperatures and minimal cross-hybridization potential (Table A1). Primers, synthesized with a 5´ C6 spacer and aminohexyl modification, were covalently conjugated by a photocleavable link to Masscode tags (Qiagen Masscode technology) (*8**,**9*). Masscode tags have a modular structure, including a tetrafluorophenyl ester for tag conjugation to primary amines; an o-nitrobenzyl photolabile linker for photoredox cleavage of the tag from the analyte; a mass spectrometry sensitivity enhancer, which improves the efficiency of atmospheric pressure chemical ionization of the cleaved tag; and a variable mass unit for variation of the cleaved tag mass (*8**,**10**–**12*). A library of 64 different tags has been established. Forward and reverse primers in individual primer sets are labeled with distinct molecular weight tags. Thus, amplification of a microbial gene target produces a dual signal that allows assessment of specificity.

Gene target standards were cloned by PCR into pCR2.1-TOPO (Invitrogen, Carlsbad, CA, USA) by using DNA template (bacterial and DNA viral targets) or cDNA template (RNA viral targets) obtained by reverse transcription of extracts from infected cultured cells or by assembly of overlapping synthetic polynucleotides. Assays were initially established by using plasmid standards diluted in 2.5-µg/mL human placenta DNA (Sigma, St. Louis, MO, USA) and subjected to PCR amplification with a multiplex PCR kit (Qiagen), primers at 0.5 µmol/L each, and the following cycling protocol: an annealing step with a temperature reduction in 1°C increments from 65°C to 51°C during the first 15 cycles and then continuing with a cycling profile of 94°C for 20 s, 50°C for 20 s, and 72°C for 30 s in an MJ PTC200 thermal cycler (MJ Research, Waltham, MA, USA). Amplification products were separated from unused primers by using QIAquick 96 PCR purification cartridges (Qiagen, with modified binding and wash buffers). Masscode tags were decoupled from amplified products through UV light-induced photolysis in a flow cell and analyzed in a single quadrapole mass spectrometer using positive-mode atmospheric pressure chemical ionization (Agilent Technologies, Palo Alto, CA, USA). A detection threshold of 100 DNA copies was determined for 19 of 22 cloned targets by using a 22-plex assay (Table 1).

**Table 1:** Sensitivity of pathogen detection by Mass Tag polymerase chain reaction determined by using plasmid and synthetic RNA standards*

Pathogen or protein	Detection threshold (DNA copies/RNA copies)
Influenza A matrix	100/1,000
Influenza A N1	100/NA
Influenza A N2	100/NA
Influenza A H1	100/NA
Influenza A H2	100/NA
Influenza A H3	100/NA
Influenza A H5	100/NA
Influenza B H	500/1,000
RSV group A	100/1,000
RSV group B	100/500
Metapneumovirus	100/1,000
CoV-SARS	100/500
CoV-OC43	100/500
CoV-229E	100/500
HPIV-1	100/1,000
HPIV-2	100/1,000
HPIV-3	100/500
*Chlamydia pneumoniae*	100/NA
*Mycoplasma pneumoniae*	100/NA
*Legionella pneumophila*	100/NA
*Enterovirus* (genus)	500/1,000
*Adenovirus* (genus)	5,000/NA

*NA, not assessed; RSV, respiratory syncytial virus; CoV, coronavirus; SARS, severe acute respiratory syndrome; HPIV, human parainfluenza virus.

Many respiratory pathogens have RNA genomes; thus, where indicated, assay sensitivity was determined by using synthetic RNA standards or RNA extracts of viral stocks. Synthetic RNA standards were generated by using T7 polymerase and linearized plasmid DNA. After quantitation by UV spectrometry, RNA was serially diluted in 2.5-µg/mL yeast tRNA (Sigma), reverse transcribed with random hexamers by using Superscript II (Invitrogen, Carlsbad, CA, USA), and used as template for Mass Tag PCR. As anticipated, sensitivity was reduced by the use of RNA instead of DNA templates (Table 1). The sensitivity of Mass Tag PCR to detect live virus was tested by using RNA extracted from serial dilutions of titered stocks of coronaviruses (severe acute respiratory syndrome [SARS] and OC43) and parainfluenzaviruses (HPIV 2 and 3). A 100-µL volume of each dilution was analyzed. RNA extracted from a 1-TCID_50_/mL dilution, representing 0.025 TCID_50_ per PCR reaction, was consistently positive in Mass Tag PCR.

**Figure 2:**
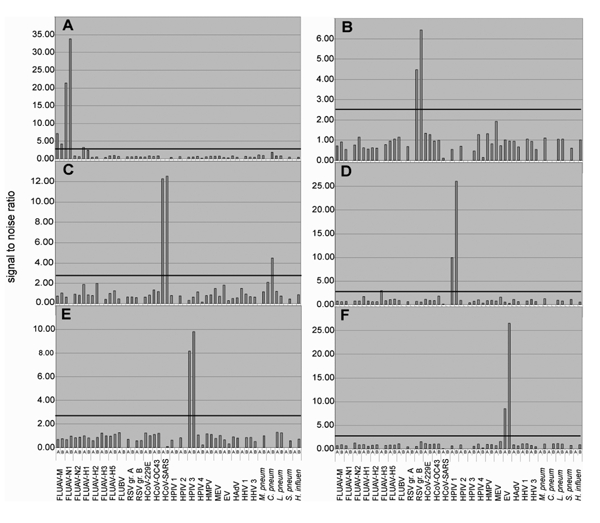
Analysis of clinical specimens. RNA extracts from clinical specimens containing known pathogens were reverse transcribed into cDNA (Superscript RT system, Invitrogen, Carlsbad, CA; 20-µL volume). Five microliters of the reaction were subjected to Mass Tag PCR by using primers coupled to Masscode tags (Qiagen Masscode technology, Qiagen, Hilden, Germany). Detection of (A) influenza virus A (H1N1), (B) respiratory syncytial virus (RSV) group B, (C) human coronavirus SARS (HCoV-SARS), (D) human parainfluenza virus (HPIV) types 1 and (E) 3, and (F) enterovirus (EV) by using a 30-plex assay, including 60 primers targeting influenza A virus matrix gene (FLUAV-M), and for typing N1, N2, H1, H2, H3, and H5 sequences, as well as influenza B virus (FLUBV), RSV groups A and B, HCoV-229E, -OC43, and -SARS, HPIV types 1, 2, 3, and 4 (groups A and B combined; 4 primers), human metapneumovirus (HMPV, 4 primers), measles virus (MEV), EV (degenerate primer pair targeting all serogroups), human adenoviruses (HAdV, degenerate primer pair targeting all serogroups), human herpesvirus 1 (HHV-1, herpes simplex virus), human herpesvirus 3 (HHV-3; varicella-zoster virus), *Mycoplasma pneumoniae*, *Chlamydia pneumoniae*, *Legionella pneumophila*, *Streptococcus pneumoniae*, *Haemophilus influenzae*. The bar indicates an arbitrary cut-off threshold of 2.7 (4 times average background determined with random human DNA).

RNA extracted from banked sputum, nasal swabs, and pulmonary washes of persons with respiratory infection was tested by using an assay panel comprising 30 gene targets that represented 22 respiratory pathogens. Infection in each of these persons had been previously diagnosed through virus isolation, conventional nested RT-PCR, or both. Reverse transcription was performed using random hexamers, and Mass Tag PCR results were consistent in all cases with the established diagnosis. Infections with respiratory syncytial virus, human parainfluenza virus, SARS coronavirus, adenovirus, enterovirus, metapneumovirus, and influenza virus were correctly identified (Table 2 and Figure 2). A panel comprising gene targets representing 17 pathogens related to central nervous system infectious disease (influenza A virus matrix gene; influenza B virus; human coronaviruses 229E, OC43, and SARS; enterovirus; adenovirus; human herpesvirus-1 and -3; West Nile virus; St. Louis encephalitis virus; measles virus; HIV-1 and -2; and *Streptococcus pneumoniae*, *Haemophilus influenzae*, and *Nesseria meningitidis*) was applied to RNA obtained from banked samples of cerebrospinal fluid and brain tissue that had been previously characterized by conventional diagnostic RT-PCR. Two of 3 cases of West Nile virus encephalitis were correctly identified. Eleven of 12 cases of enteroviral meningitis were detected representing serotypes CV-B2, CV-B3, CV-B5, E-6, E-11, E-13, E-18, and E-30 (data not shown).

**Table 2:** Multiplex pathogen detection by Mass Tag polymerase chain reaction using Masscode-labeled primers in a 30-plex assay with clinical specimens with previously identified pathogens*

Pathogen	No. positive/no. tested†
RSV A	2/2
RSV B	3/3
HPIV-1	1/1
HPIV-3	2/2
HPIV-4	2/2
CoV-SARS	4/4
Metapneumovirus	2/3
Influenza B	1/3
Influenza A	2/6
Adenovirus	2/2
Enterovirus	2/2

*RSV, respiratory syncytial virus; HPIV, human parainfluenza virus; CoV, coronavirus; SARS, severe acute respiratory syndrome. †No. positive and consistent with previous diagnosis/number tested (with respective previous diagnosis).

## Conclusions

Our results indicate that Mass Tag PCR is a sensitive and specific tool for molecular characterization of microflora. The advantage of Mass Tag PCR is its capacity for multiplex analysis. Although the use of degenerate primers (e.g., enteroviruses and adenoviruses, Table A1 and Table 1) may reduce sensitivity, the limit of multiplexing to detect specific targets will likely be defined by the maximal primer concentration that can be accommodated in a PCR mix. Analysis requires the purification of product from unincorporated primers and mass spectroscopy. Although these steps are now performed manually, and mass spectrometers are not yet widely distributed in clinical laboratories, the increasing popularity of mass spectrometry in biomedical sciences and the advent of smaller, lower-cost instruments could facilitate wider use and integrated instrumentation. In addition to developing additional pathogen panels, our continuing work is focused on optimizing multiplexing, sensitivity, and throughput. Potential applications include differential diagnosis of infectious diseases, blood product surveillance, forensic microbiology, and biodefense.

## Appendix

**Table A1:** Respiratory panel Mass Tag primers.

Target – Masscode FWD/REV	Name FWD	Sequence	Name REV	Sequence	Ref.
FLUAV-M 618/690	AM-U151	CATGGAATGGCTAAAGACAAGACC	AM-L397	AAGTGCACCAGCAGAATAACTGAG	(*8*)
FLUAV-N1 499/439	NA1-U1078	ATGGTAATGGTGTTTGGATAGGAAG	NA1-L1352	AATGCTGCTCCCACTAGTCCAG	(*8*)
FLUAV-N2 658/730	NA2-U560	AAGCATGGCTGCATGTTTGTG	NA2-L858	ACCAGGATATCGAGGATAACAGGA	(*8*)
FLUAV-HA1 650/590	HA1-U583	GGTGTTCATCACCCGTCTAACAT	HA1-L895	GTGTTTGACACTTCGCGTCACAT	(*8*)
FLUAV-HA2 662/539	H2A208U27	GCTATGCAAACTAAACGGAATYCCTCC	H2A559L26	TATTGTTGTACGATCCTTTGGCAACC	
FLUAV-HA3-1 586/475	HA3-U115	GCTACTGAGCTGGTTCAGAGTTC	HA3-L375	GAAGTCTTCATTGATAAACTCCAG	(*8*)
FLUAV-HA5 646/395	HA5hum-u71	TTACTGTTACACATGCCCAAGACA	HA5hum-L147	AGGYTTCACTCCATTTAGATCGCA	
FLUBV 698/598	BHA-U188	AGACCAGAGGGAAACTATGCCC	BHA-L347	CTGTCGTGCATTATAGGAAAGCAC	(*8*)
HCoV-SARS 527/666	CIID-28891F	AAGCCTCGCCAAAAACGTAC	CIID-29100R	AAGTCAGCCATGTTCCCGAA	
HCoV-229E 670/558	Taq-Co22-418F	GGCGCAAGAATTCAGAACCA	Taq-Co22-636R	TAAGAGCCGCAGCAACTGC	
HCoV-OC43 686/548	Taq-Co43-270F	TGTGCCTATTGCACCAGGAGT	Taq-Co43-508R	CCCGATCGACAATGTCAGC	
RSV A 467/455	RSA-U1137	AGATCAACTTCTGTCATCCAGCAA	RSV-L1192	GCACATCATAATTAGGAGTATCAAT	(*9*)
RSV B 483/479	RSB-U1248	AAGATGCAAATCATAAATTCACAGGA	RSV-1318	TGATATCCAGCATCTTTAAGTATCTTTATAGTG	(*9*)
HMPV-1 718/654	MPV01.2	AACCGTGTACTAAGTGATGCACTC	MPV02.2	CATTGTTTGACCGGCCCCATAA	(*10*)
HMPV-2 718/654	MV-Can-U918	AAGTCCAAAGGCAGGRCTGTTATC	MV-Can-L992	CCTGAAGCATTRCCAAGAACAACAC	
HPIV-1 566/357	HPIV1-U82	TACTTTTGACACATTTAGTTCCAGGAG	HPIV1-L167	CGGTACTTCTTTGACCAGGTATAATTG	
HPIV-2 463/590	HPIV2-U908	GGACTTGGAACAAGATGGCCT	HPIV2-L984	AGCATGAGAGCYTTTAATTTCTGGA	
HPIV-3 423/539	HPIV3-U590	GCTTTCAGACAAGATGGAACAGTG	HPIV3-L668	GCATKATTGACCCAATCTGATCC	
HPIV 4A 622/606	HPIV4A-U191	AACAGAAGGAAATGATGGTGGAAC	HPIV4A-L269	TGCTGTGGATGTATGGGCAG	
HPIV 4B 622/606	HPIV4B-U194	AGAAGAAAACAACGATGAGACAAGG	HPIV4B-L306	GTTTCCCTGGTTCACTCTCTTCA	
Measles 578/562	MEA-U1103	CAAGCATCATGATYGCCATTCCTGG	MEA-L1183	CCTGAATCYCTGCCTATGATGGGTTT	
Adenovirus 503/630	ADV2HEX2F	CCCMTTYAACCACCACCG	ADVHEX1R	ACATCCTTBCKGAAGTTCCA	(*11*)
Enterovirus 702/495	5UTR-U447	TCCTCCGGCCCCTGAATGCGGCTAATCC	5UTR-L541	GAAACACGGWCACCCAAAGTASTCG	
HHV-1 443/706	HSV-U27	CCCGGATGCGGTCCAGACGATTAT	HSV-L121	CCCGCGGAGGTTGTACAAAAAGCT	
HHV-3 515/471	VZV-U138	ACGTGGATCGTCGGATCAGTTGT	VZV-L196	TCGCTATGTGCTAAAACACGCGG	
*Legionella pneumophila* 678/582	LegPneu-U149	GCATWGATGTTARTCCGGAAGCA	LegPneu-L223	CGGTTAAAGCCAATTGAGCG	
*Chlamydia pneumoniae* 519/383	CLPM1	CATGGTGTCATTCGCCAAGT	CLPM2	CGTGTCGTCCAGCCATTTTA	(*12*)
*Streptococcus pneumonia* 714/694	SPPLY-U532	AGCGATAGCTTTCTCCAAGTGG	SPPLY-L606	CTTAGCCAACAAATCGTTTACCG	
*Haemophilus influenzae* 734/726	HINF-U82	AAGCTCCTTGMATTTTTTGTATTAGAA	Hinf-L158	GCTGAATTGGCTTRGATACCGAG	
*Mycoplasma pneumoniae* 602/614	MTPM1	CCAACCAAACAACAACGTTCA	MTPM2	ACCTTGACTGGAGGCCGTTA	(*12*)
